# First-Principles Study of Interfacial Properties and Fracture Behavior of (3C and 4H) SiC/Al Interfaces

**DOI:** 10.3390/ma19081536

**Published:** 2026-04-12

**Authors:** Rong Zhang, Yongbiao Zhong, Kaile Zhao, Junfeng Wang, Junhui Si, Yuping Wu, Chunming Zou, Hongwei Wang, Zunjie Wei

**Affiliations:** 1 School of Materials Science and Engineering, Fujian University of Technology, Fuzhou 350118, Chinasijunhui2004@126.com (J.S.); wyping1022@163.com (Y.W.); 2 National Key Laboratory for Precision Hot Processing of Metals, Harbin Institute of Technology, Harbin 150001, China

**Keywords:** SiC/Al interfaces, work of separation, tensile fracture behavior, electronic structure, first-principles calculation, density functional theory, metal–ceramic composites

## Abstract

First-principles calculations based on density functional theory (DFT) are performed to investigate the interfacial properties and fracture behavior of 3C-SiC(111)/Al(111) and 4H-SiC(0001)/Al(111) interfaces. To mitigate surface effects through adequate slab thickness, the interface models are constructed by positioning a seven-layer Al(111) slab atop eight-layer 3C-SiC(111) and 14-layer 4H-SiC(0001) slabs, respectively. Accounting for the distinct surface terminations and stacking sequences of each polytype, six interface configurations are established: C-top, -center, and -hollow; Si-top, -center, and -hollow. Based on the simulation results of surface energy, work of separation, and electron density distribution, the C-top configuration yields the most stable SiC/Al interface structure, exhibiting the highest work of separation. The ultimate tensile strengths of the C-top interfaces are 6.603 GPa (3C-SiC/Al) and 6.851 GPa (4H-SiC/Al), with corresponding tensile strains of 10% and 12%, respectively. Tensile fracture initiates exclusively within the Al slab for all C-top interfaces, but at distinct atomic layers: fracture occurs between the second and third Al layers (Al2–Al3) for 3C-SiC/Al; and between the first and second Al layers (Al1–Al2) for 4H-SiC/Al. This distinction reflects the influence of different interfacial configurations on the bonding strength between aluminum atomic layers. In summary, an atomic-scale investigation of the interfacial properties and fracture behavior of SiC/Al interfaces provides critical insights for the design and fabrication of novel ceramic/metal composites.

## 1. Introduction

Advances in aerospace and electronic communication technologies continue to drive the need for materials with enhanced performance. An ideal composite should offer not only high-temperature and wear resistance but also superior dimensional and chemical stability. Silicon carbide-reinforced aluminum (SiC/Al) composites have gained significant attention as a promising structural material, owing to their attractive combination of properties: low density, high specific strength and stiffness, low thermal expansion coefficient, high thermal conductivity, good corrosion and wear resistance, along with ease of processing and low cost. These advantages have led to their widespread application and in-depth research in recent years [1,2,3,4,5,6]. It is essential to elucidate the differences in the mechanical responses of interfaces formed by different SiC crystal structures and the aluminum matrix under external loading. Such understanding is crucial for optimizing material design according to specific service conditions, rationally selecting SiC polytypes and composite systems, and thereby ensuring the safety and reliability of components in practical applications. Among the more than 250 SiC crystal structures [7], several are particularly notable: 3C-SiC offers high strength, hardness, and wear resistance [8,9]; 4H-SiC features a high melting point, strength, thermal conductivity, mechanical stability, stiffness, and power density, ideal for high-power and high-temperature electronic devices [10,11]; and 6H-SiC, with its wide bandgap and high thermal/mechanical stability, suits applications ranging from light-emitting devices to high-durability mechanical parts [12,13].

The influence of temperature, chemical composition, crystal structure, atomic/electronic interactions, and processing conditions on material properties is significant [14,15]. At the microstructural scale, the distinct atomic arrangements of various SiC polytypes lead to differences in chemical bonding parameters like energy, length, and direction. These differences affect the interfacial properties between SiC and Al, thereby influencing the macroscopic mechanical properties of the material.

First-principles calculations are extensively employed to elucidate the atomic structure and electronic properties of interfaces in metal matrix composites. These computations can facilitate the prediction and analysis of the energy and structure of interfaces between heterogeneous nucleants and primary phases in solidification processes [16,17]. It was reported that the bonding strength at the interface with the metallic matrix is significantly influenced by the crystal structure of SiC (cubic or hexagonal) and its surface termination (C-terminated or Si-terminated). As an example, Xu et al. [18] discovered through first-principles calculations that the C-terminated surface of 4H-SiC(0001) displays greater reactivity than its Si-terminated counterpart. The bonding strength at the C-terminated 4H-SiC(0001)/Al(111) interface is substantially enhanced by the introduction of Ti and Si. Qiu et al. [19] investigated the β-SiC(111)/Al(111) interface, finding that the C-terminated ‘top’ stacking configuration exhibited the highest interfacial chemical energy and tensile strength (6.33 GPa). Furthermore, research by Wu et al. [20] on 6H-SiC(0001)/Al(111) interfaces indicated that bonding energy for C-terminated interfaces (2.689 J/m^2^) exceeded that of Si-terminated interfaces (1.649 J/m^2^). Through comparative analysis of the interfacial energies between 3C-SiC and 6H-SiC with Al, Zhang et al. [21] further demonstrated that the C-terminated interface exhibits greater thermodynamic stability owing to its stronger covalent bonding characteristics.

Although considerable research has been conducted on SiC/Al composites, a systematic investigation into the failure mechanisms of interfaces between different SiC polytypes (such as 3C- and 4H-SiC) and Al under external loading is still lacking. 3C-SiC is often associated with favorable mechanical properties, whereas 4H-SiC is widely used in high-temperature and electronic applications. Their different crystal structures (cubic vs. hexagonal stacking) may influence interfacial bonding and mechanical behavior differently. To address this, the present study employs first-principles calculations to methodically investigate the atomic structure, tensile deformation behavior, and failure mechanisms at the interfaces between various SiC polytypes and Al. This research provides a theoretical basis for a deeper understanding of the interfacial bonding mechanisms and the performance-optimized design of such composite materials. Transmission electron microscopy (TEM) observations of metal/SiC interfaces would be used to validate the simulation results in future work.

## 2. Calculation Methodology

First-principles calculations based on density functional theory (DFT) were conducted with the Cambridge Sequential Total Energy Package (CASTEP) in Materials Studio 2017 R2 software [22,23]. This method can accurately describe the electronic structure and the interaction between atoms by solving the Kohn–Sham equation, which is suitable for the study of the interface system of metal matrix composites [24]. The Perdew–Burke–Ernzerhof (PBE) functional within the generalized gradient approximation (GGA) was employed for describing the exchange–correlation energy [25]. Based on convergence tests, the plane-wave cutoff energy was set at 600 eV to ensure sufficient computational accuracy. The Si (3s^2^ 3p^2^), C (2s^2^ 2p^2^), and Al (3s^2^ 3p^1^) were treated as valence electrons. A Monkhorst–Pack k-point sampling scheme was employed [26]. Effects such as spin polarization and van der Waals corrections were not included in the calculations. For the bulk materials, k-point meshes of 6 × 6 × 6 were employed for Al and 3C-SiC, and 5 × 5 × 2 for 4H-SiC. For the interfacial systems, k-point meshes of 15 × 15 × 1 and 10 × 10 × 1 were adopted for the 3C- and 4H-SiC/Al interfaces, respectively. The convergence tolerances were set to 1.0 × 10^−5^ eV/atom for energy, 0.03 eV/Å for force, 0.001 Å for atomic displacement, and 0.05 GPa for stress.

**Figure 1:**
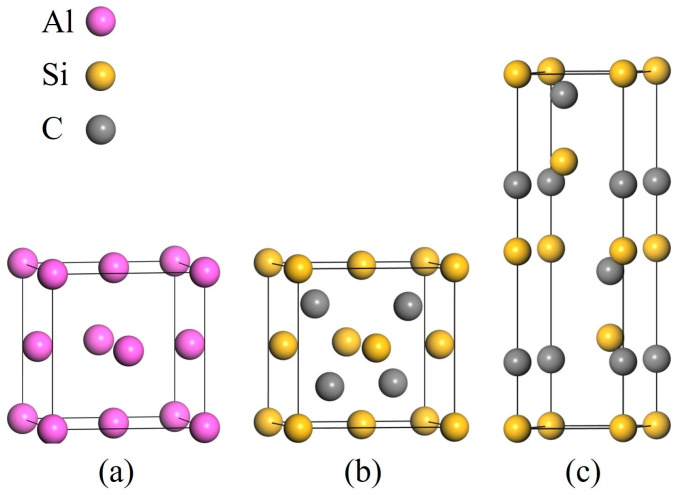
Crystal structures: (**a**) Al; (**b**) 3C-SiC; (**c**) 4H-SiC.

The crystal structure models employed in this study are presented in Figure 1. Al and 3C-SiC are modeled with face-centered cubic (FCC) structures, while 4H-SiC is modeled with hexagonal close-packed (HCP) structures. The corresponding space groups are Fm-3m (No. 225) for Al, F-43m (No. 216) for 3C-SiC, and P63mc (No. 186) for 4H-SiC.

To assess the accuracy of the computational methodology employed in this work, a comparison was made between the calculated lattice constants of Al and SiC and the results obtained from different theoretical methods (GGA-PBE, GGA-PW91, and LDA-CAPZ) as well as experimental values, as shown in Table 1. The good agreement between the calculated results and the reference data demonstrated the rationality and reliability of the computational method employed in this work.

## 3. Results and Discussion

### 3.1. Surface Structure and Energy

**Figure 2:**
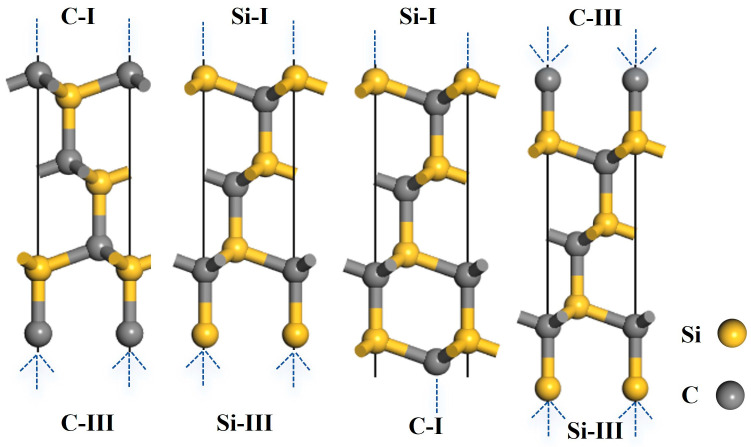
Structural models of the 3C-SiC(111) slabs.

**Figure 3:**
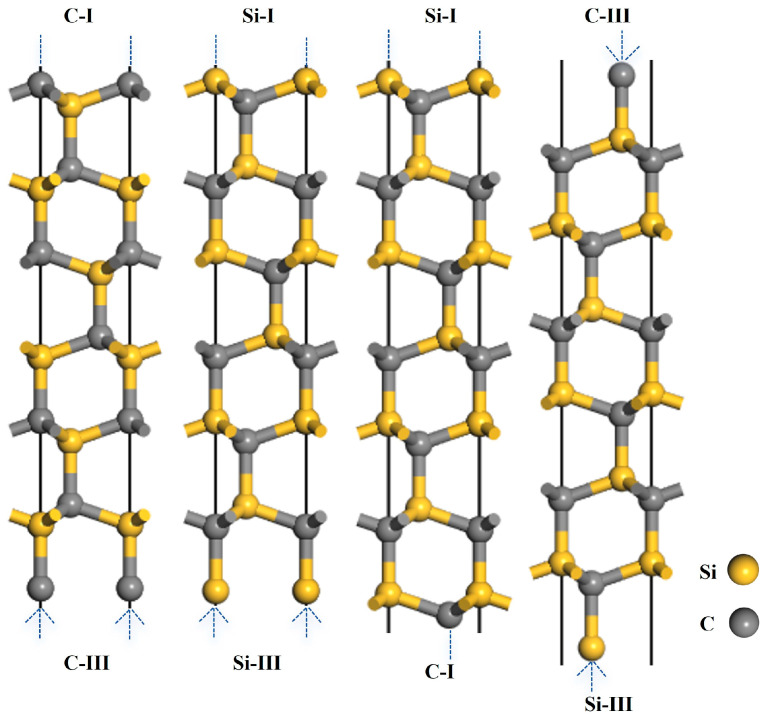
Structural models of the 4H-SiC(0001) slabs.

This study investigated typical low-index SiC surfaces, namely 3C-SiC(111) and 4H-SiC(0001). Their atomic structures are presented in Figure 2 and Figure 3. Each surface exhibited two types of polar terminations: the Si-terminated surface and the C-terminated surface. Based on the number of dangling bonds, these terminations were further categorized into two distinct bonding configurations: the Si-I (or C-I) surface with a single dangling bond, and the Si-III (or C-III) surface with three dangling bonds. Structural relaxation was performed on C- and Si-terminated slabs with varying numbers of layers to obtain stable surfaces for 3C-SiC(111) and 4H-SiC(0001). A 15 Å vacuum layer was applied to all models to prevent spurious periodic interactions. The optimal number of layers was determined by achieving convergence in the interlayer distances before and after relaxation. The relative change in the interlayer spacing was calculated using the following equation [34]:

(1) $$∆d_{ij}=\frac{d_{ij}-d_{0}}{d_{0}}\times 100\%$$

where *d_ij_* and *d*_0_ are the interlayer distances between the *i*-th and *j*-th layers for the relaxed structure and the bulk, respectively. A positive (negative) value signifies interlayer expansion (contraction).

Structural relaxation was performed on the Si- and C-terminated surface models of 3C–SiC(111), and the results are summarized in Table 2. When the number of layers reached seven or more, the relative change in interlayer spacing between the outermost and sub-outermost layers of the Si–I and C–I surfaces converged, indicating that the slab thickness was sufficient to represent bulk properties. Due to the asymmetric distribution of dangling bonds on the upper and lower sides of odd-layered 3C–SiC(111) surfaces, an eight-layer even-numbered slab was adopted for subsequent calculations. This model ensures the stoichiometric ratio of the 3C-SiC slab.

To investigate the influence of the number of dangling bonds on surface energy, the surface energies of two terminations (Si–III/C–III and Si–I/C–I) were calculated using their respective models. The eight-layer periodic slab calculations employed the non-self-consistent dipole correction method [35]. The results indicate that the surface energy for the configuration with three dangling bonds on both sides is 6.996 J/m^2^, whereas that with one dangling bond on both sides is 4.092 J/m^2^. Since a lower surface energy corresponds to higher stability, the configuration with one dangling bond on each side is more stable. Therefore, we constructed the subsequent interface models using the 3C-SiC(111) surface with one dangling bond on both the upper and lower terminations.

Structural relaxation was performed on the Si- and C-terminated surface models of 4H–SiC(0001), and the results are summarized in Table 3. When the number of layers reached thirteen or more, the relative change in interlayer spacing between the outermost and sub-outermost layers of the Si–I and C–I surfaces converged, indicating that the slab thickness was sufficient to represent bulk properties. Due to the asymmetric distribution of dangling bonds on the upper and lower sides of odd-layered 4H–SiC(0001) surfaces, a 14-layer even-numbered slab was adopted for subsequent calculations. This model ensures the stoichiometric ratio of the 4H-SiC slab.

The surface energy of 4H-SiC(0001) under different numbers of dangling bonds was calculated. The 14-layer periodic slab calculations employed the non-self-consistent dipole correction method [35]. The results indicate that the surface energy for the configuration with three dangling bonds on both sides is 6.931 J/m^2^, whereas that with one dangling bond on both sides is 4.160 J/m^2^. Due to higher stability indicated by a lower surface energy, the 4H-SiC(0001) surface with a single dangling bond on each termination was selected for constructing the interface models.

The stability of a surface was evaluated by its surface energy, since a lower value corresponds to a more stable structure. The surface energy formulae for different crystal planes are given below

(2) $$E_{surf}=\frac{E_{slab}-nE_{bulk}}{2A}$$

where *E*_slab_ and *E*_bulk_ are the energies of the relaxed slab and bulk crystal, respectively, *n* is the ratio of the atom count in the slab to that in the bulk crystal, and *A* is the surface area, given as *A* = a × b × sin(γ/180 × π).

In this study, low-index Al(001), Al(110), and Al(111) surfaces were selected to construct slab models with a 15 Å vacuum layer. After structural optimization of these surfaces, their surface energies were calculated. As shown in Table 4, our calculated results are consistent with other theoretical values, confirming that the Al(111) surface possesses the lowest surface energy and thus is the most stable. Therefore, the Al(111) surface was chosen for subsequent interface construction.

**Figure 4:**
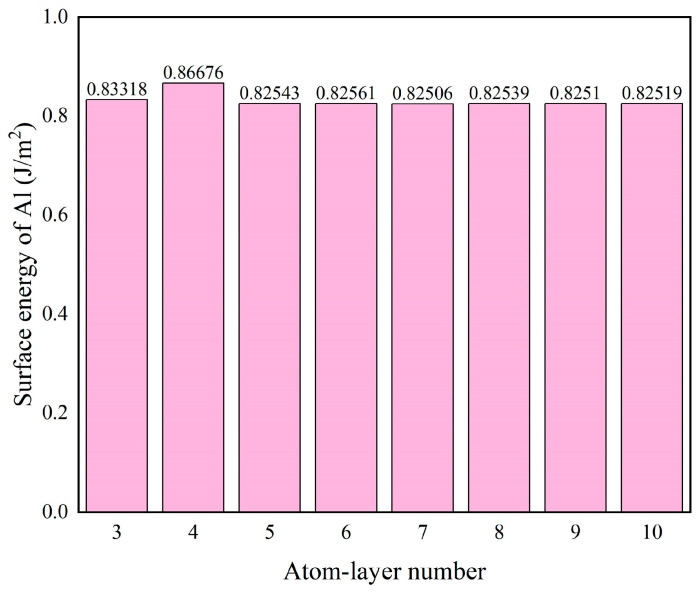
Surface energy of the Al(111) surface as a function of atomic layer number.

To determine an appropriate slab thickness, a convergence test of the Al(111) surface energy with respect to the number of atomic layers was performed. As shown in Figure 4, the energy converges for slabs thicker than five layers.

### 3.2. Construction of the Interface Model

**Figure 5:**
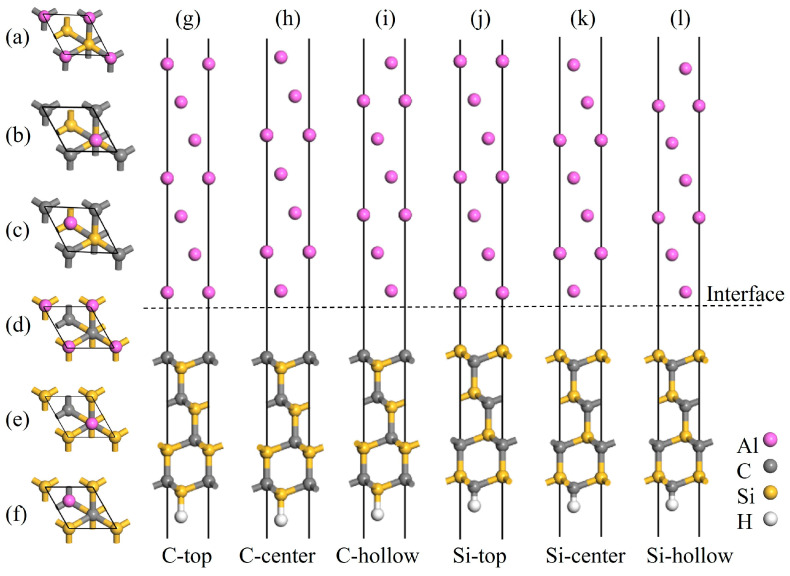
Atomic structures of the C- and Si-terminated 3C-SiC(111)/Al(111) interface models: (**a**,**g**) C-top site, (**b**,**h**) C-center site, (**c**,**i**) C-hollow site, (**d**,**j**) Si-top site, (**e**,**k**) Si-center site, and (**f**,**l**) Si-hollow site.

**Figure 6:**
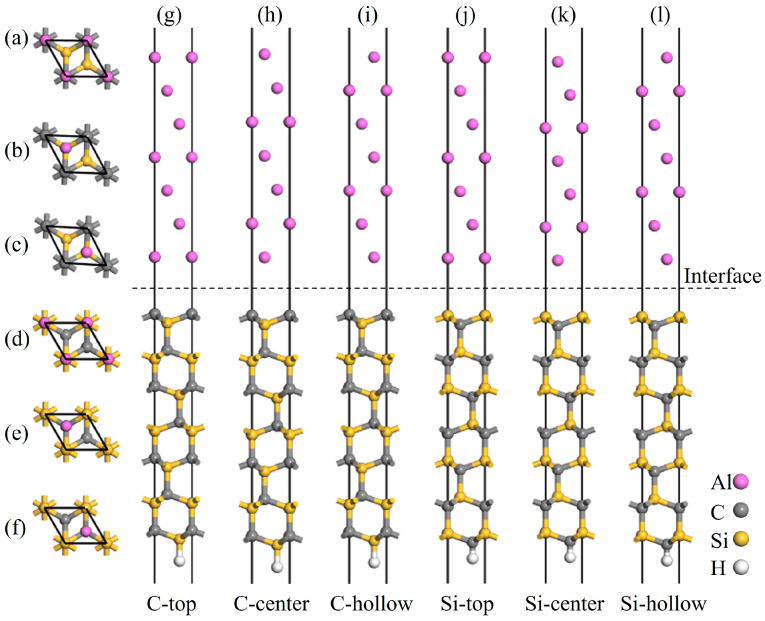
Atomic structures of the C- and Si-terminated 4H-SiC(0001)/Al(111) interface models: (**a**,**g**) C-top site, (**b**,**h**) C-center site, (**c**,**i**) C-hollow site, (**d**,**j**) Si-top site, (**e**,**k**) Si-center site, and (**f**,**l**) Si-hollow site.

The surface energy of Al(111) converges beyond a thickness of five atomic layers. However, in interfacial tensile simulations, it is necessary to fix the bottom-layer atoms to simulate bulk constraints. Moreover, lattice mismatch at the Al/SiC interface is accommodated primarily through deformation of the aluminum layers. Thus, two additional Al layers were added beyond the five-layer configuration to better satisfy boundary constraints and allow adequate room for interfacial relaxation. Based on the previously determined optimal slab thicknesses, the interface models were constructed by positioning a seven-layer Al(111) slab atop the 3C-SiC(111) (eight-layer) and 4H-SiC(0001) (14-layer) slabs. The consideration of two terminations and three stacking sequences for the SiC slabs results in a total of six models to be calculated. As shown in Figure 5 and Figure 6, the Al atoms are positioned directly above the first-layer SiC atoms in the C-top and Si-top configurations; directly above the second-layer SiC atoms in the C-center and Si-center configurations; and above the hollow sites of the SiC surface in the C-hollow and Si-hollow configurations. To ensure computational accuracy, a 15 Å vacuum layer was applied to all interface models to prevent spurious periodic interactions. Spurious dipole moments were eliminated by saturating the bottom-layer C or Si atoms with H atoms during the model construction [37].

### 3.3. Interfacial Distance and Work of Separation

The work of separation (*W*_sep_), defined as the reversible work needed to separate an interface into two free surfaces without relaxation, is a key quantity for predicting interfacial strength. A direct correlation exists between the magnitude of *W*_sep_ and the interfacial bonding strength. *W*_sep_ can be expressed as follows [38,39]:

(3) $$W_{sep}=\frac{\left(E_{Al}+E_{SiC}-E_{Al/SiC}\right)}{A}$$

where *E*_Al/SiC_ is the energy of the interface model; *E*_Al_ and *E*_SiC_ are the energies of the Al and SiC separated slabs, respectively; and *A* is the interfacial area.

**Figure 7:**
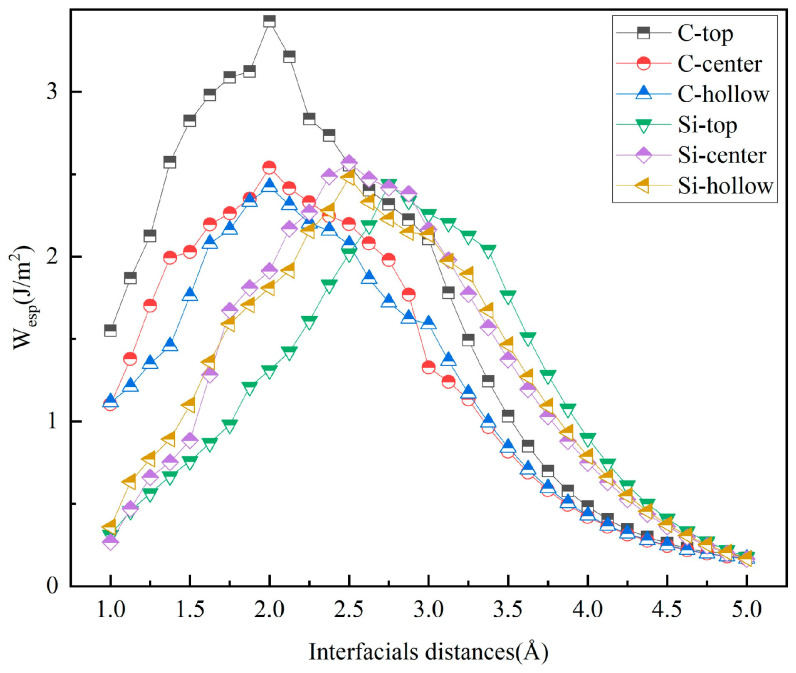
Work of separation (*W*_sep_) as a function of interfacial distance (*d*_0_) for the unrelaxed 3C-SiC(111)/Al(111) interfaces.

**Figure 8:**
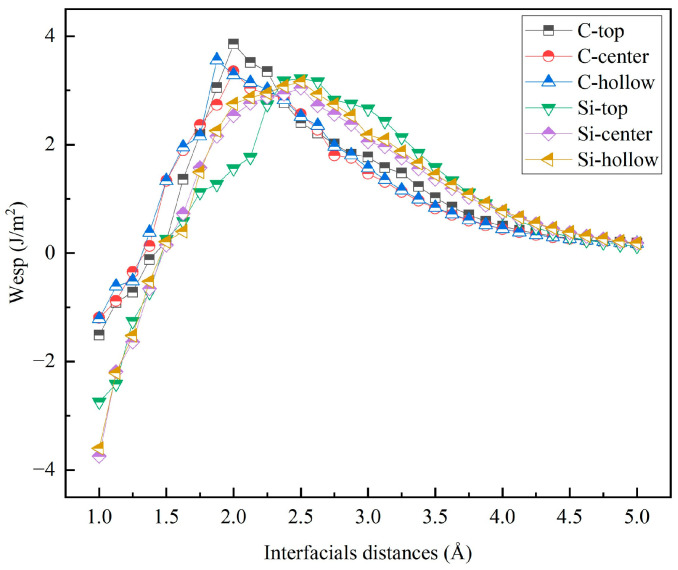
Work of separation (*W*_sep_) as a function of interfacial distance (*d*_0_) for the unrelaxed 4H-SiC(0001)/Al(111) interfaces.

The judicious selection of the initial interfacial distance is paramount for optimizing computational efficiency and ensuring result accuracy. In this study, *W*_sep_ was systematically calculated for various initial interfacial distances using the Universal Binding Energy Relationship (UBER) method [37]. Figure 7 and Figure 8 present the UBER curves of *W*_sep_ as a function of the interfacial distance for the unrelaxed interface models. The peak position on each curve indicates the maximum *W*_sep_ and the corresponding interfacial distance, with the specific values listed in the “Unrelaxed” columns of Table 5 and Table 6. The results reveal that *W*_sep_ initially increases with the interfacial distance, reaches a maximum, and then gradually decreases, with this peak position corresponding to the highest interfacial bonding strength.

Subsequently, interface models were constructed based on the interfacial distances predicted by the UBER and subjected to structural relaxation. This process yielded the equilibrium interfacial distances and *W*_sep_, with the specific values listed in the “Relaxed” columns of Table 5 and Table 6. The computational data reveal that among the 3C-SiC(111)/Al(111) and 4H-SiC(0001)/Al(111) interfaces, the C-top stacking configuration exhibits the highest *W*_sep_ values of 3.640 J/m^2^ and 3.971 J/m^2^, respectively. This indicates that the C-top configuration possesses the strongest interfacial bonding strength.

In the construction of the interface models, SiC was used as the substrate, with the Al layer being strained to match the lattice constant of SiC. The initial lattice mismatch values for the 3C-SiC(111)/Al(111) and 4H-SiC(0001)/Al(111) interfaces were calculated to be 7.62% and 7.59%, respectively. After geometry optimization, the in-plane lattice constants of the supercell were determined, from which the residual strains in the Al and SiC layers, relative to their respective bulk equilibrium lattice constants, were calculated. The results indicate that the residual tensile strains in the Al layer are 6.71% and 7.2% for the 3C-SiC/Al and 4H-SiC/Al interfaces, respectively, while the corresponding compressive strains in the SiC layer are 1.42% and 0.94%. This demonstrates that significant residual tensile strain is introduced into the Al upon interface formation, which is likely to influence its mechanical behavior. These computational results are consistent with the actual physical scenario: in real composites, owing to the relatively large difference in the coefficients of thermal expansion between Al and SiC, macroscopic residual tensile stress develops in the Al matrix upon cooling from the processing temperature to room temperature.

### 3.4. Electronic Structure

**Figure 9:**
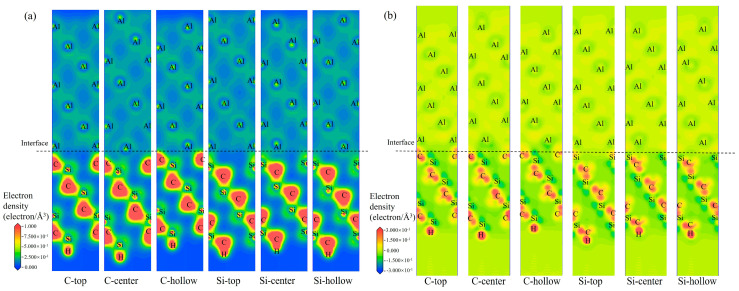
Electronic properties at the 3C-SiC(111)/Al(111) interface for six interfacial configurations: (**a**) valence electron density distribution; (**b**) charge density difference.

**Figure 10:**
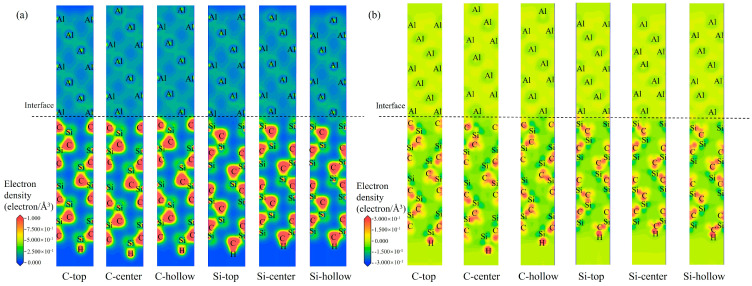
Electronic properties at the 4H-SiC(0001)/Al(111) interface for six interfacial configurations: (**a**) valence electron density distribution; (**b**) charge density difference.

Figure 9 and Figure 10 show the valence electron density and charge density difference for the different interfacial configurations (C-top, -center, and -hollow; Si-top, -center, and -hollow) at the 3C-SiC(111)/Al(111) and 4H-SiC(0001)/Al(111) interfaces, respectively. Comparative analysis of the valence electron density distributions in Figure 9a and Figure 10a reveals that the C-terminated surface exhibits higher electron density at the interface with Al compared to the Si-terminated surface, indicating stronger interfacial interaction between the C-terminated surface and Al. As shown in Figure 9b and Figure 10b, among the SiC/Al interfaces, the C-top model exhibits the most pronounced variation in charge density. A larger positive charge density difference reflects more significant charge transfer in that region, leading to stronger charge interaction.

To further investigate the electron distribution and bonding interactions at the 3C-SiC(111)/Al(111) and 4H-SiC(0001)/Al(111) interfaces, the calculated Mulliken population results for interfacial atoms in both the C- and Si-top configurations are summarized in Table 7 and Table 8.

In the C-top 3C-SiC(111)/Al(111) interface, the interfacial Al atom exhibits a significant charge loss of 0.28 e. It forms a bond with the C atom, as indicated by a bond population of 0.25. In contrast, for the Si-top 3C-SiC(111)/Al(111) interface, the Al atom gains a small amount of charge (0.05 e) and is bonded to a Si atom, with a bond population of 0.33.

A similar trend is observed for the 4H-SiC(0001)/Al(111) interface. With the C-top termination, the Al atom shows a charge loss of 0.28 e and a bond population of 0.26 with the C atom. Conversely, with the Si-top termination, the Al atom gains 0.05 e and exhibits a bond population of 0.35 with the Si atom.

The integration of interface spacing, work of separation, and electronic structure analysis indicates that the C-top configuration has the highest interfacial bonding strength. Based on this conclusion, subsequent tensile mechanical property analyses of the interfacial systems are conducted exclusively using the C-top model.

### 3.5. Tensile Test of the SiC/Al Interface

To investigate the tensile properties of the SiC/Al interface with the C-top configuration, the relaxed-type tensile method was employed. In these calculations, the bottom-layer atoms were fixed, and strain was applied along the respective crystallographic directions ([111] for 3C-SiC and [0001] for 4H-SiC) to determine the ideal tensile strength and analyze the interfacial failure mode. During the construction of the interface model, the z-axis of the Cartesian coordinate system was strictly aligned with the tensile direction ([111] or [0001]). Thus, the σ_zz_ component of the stress tensor represents the normal tensile stress along that crystallographic direction.

**Figure 11:**
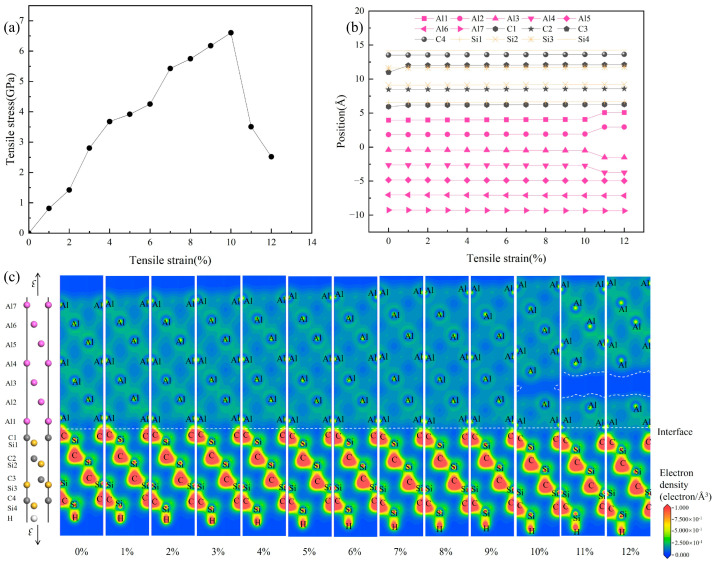
Tensile failure process of the 3C-SiC(111)/Al(111) interface (C-top configuration): (**a**) stress–strain curves; (**b**) evolution of atomic positions with strain; (**c**) evolution of valence electron density with strain.

Figure 11 presents the evolution of (a) the tensile stress–strain curve, (b) the atomic positions, and (c) valence electron density distributions for the 3C-SiC/Al interface under tensile loading, which are used to analyze its deformation behavior and fracture mechanism. The stress–strain relationship in Figure 11a shows that the interface model has a tensile strength of 6.603 GPa and a fracture elongation of 10%. It should be noted that the ideal strength obtained from simulations is typically higher than experimental values, as pre-existing cracks and microstructural defects in real materials significantly degrade their mechanical properties. Figure 11b displays the positional changes in the Si, C, and Al atoms as the strain increases from 0% to 12%. When the strain exceeds 10%, the stress begins to drop, accompanied by a significant increase in the spacing between the second and third aluminum layers (Al2–Al3), indicating that fracture initiates in this region. To further reveal the electronic structure evolution during fracture, Figure 11c shows the valence electron density maps at various strain levels. The results indicate that the valence electron density distribution remains essentially unchanged within the 0–9% strain range. At 10% strain, partial fracture occurs between Al2 and Al3. As the strain increases beyond 10%, the fractured area expands across the entire interlayer region between the aluminum layers. The area enclosed by the white dashed lines exhibits minimal valence electron density, clearly revealing the precise location of fracture initiation at the interface during tensile deformation.

**Figure 12:**
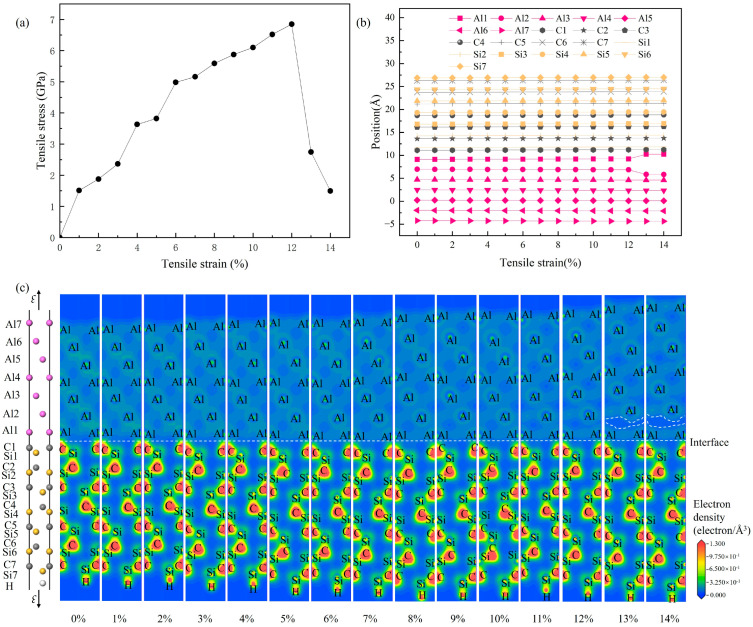
Tensile failure process of the 4H-SiC(0001)/Al(111) interface (C-top configuration): (**a**) stress–strain curves; (**b**) evolution of atomic positions with strain; (**c**) evolution of valence electron density with strain.

Figure 12 presents the evolution of (a) the tensile stress–strain curve, (b) the atomic positions, and (c) valence electron density distributions for the 4H-SiC/Al interface under tensile loading, which are used to analyze its deformation behavior and fracture mechanism. The stress–strain relationship in Figure 12a shows that the interface model has a tensile strength of 6.851 GPa and a fracture elongation of 12%. Figure 12b displays the positional changes in the Si, C, and Al atoms as the strain increases from 0% to 14%. When the strain exceeds 12%, the stress begins to drop, accompanied by a significant increase in the spacing between the first and second aluminum layers (Al1–Al2), indicating that fracture initiates in this region. To further reveal the electronic structure evolution during fracture, Figure 12c shows the valence electron density maps at various strain levels. The results indicate that the valence electron density distribution remains essentially unchanged within the 0–12% strain range. As the strain increases beyond 12%, the fractured area expands across the entire interlayer region between the aluminum layers. The area enclosed by the white dashed lines exhibits minimal valence electron density, clearly revealing the precise location of fracture initiation at the interface during tensile deformation.

This work investigates and compares the tensile properties of two SiC/Al interface models using first-principles calculations. The results indicate that the 4H-SiC/Al interface exhibits the highest ultimate tensile strength (6.851 GPa) and ultimate tensile strain (12%), with fracture occurring at the Al1-Al2 interface. In contrast, the 3C-SiC/Al interface has a lower ultimate tensile strain of 10%, and fracture initiates at the Al2-Al3 interface. This distinction reflects the influence of different interfacial configurations on the bonding strength between aluminum atomic layers.

Table 9 presents a comparison of the key parameters (work of separation, ultimate tensile strength, and ultimate tensile strain) obtained from first-principles simulations in this study with the literature data. The results indicate that the 4H-SiC(0001)/Al(111) interface possesses the highest ultimate tensile strength and ultimate tensile strain.

The interfaces considered in this study are ideal, without defects or impurities. However, real composite interfaces typically contain such features, which influence their mechanical behavior. As shown in Table 10, the measured tensile strengths of the 9–25vol%SiCp/2009Al composites are lower than the calculated tensile strengths.

## 4. Conclusions

This study employs first-principles calculations based on density functional theory to systematically investigate the atomic structure, tensile deformation, and failure mechanisms of interfaces: 3C-SiC(111)/Al(111) and 4H-SiC(0001)/Al(111). The main findings are summarized as follows:

(1) To mitigate surface effects through adequate slab thickness, the interface models are constructed by positioning a seven-layer Al(111) slab atop eight-layer 3C-SiC(111) and 14-layer 4H-SiC(0001) slabs, respectively. Among the six interfacial configurations (C-top, -center, and -hollow; Si-top, -center, and -hollow), the C-top configuration consistently exhibited the highest work of separation across all SiC/Al interfaces. Consequently, it was selected as the representative model for subsequent tensile property investigations.
(2) Tensile loading was applied along the [111] direction for 3C-SiC and the [0001] direction for 4H-SiC until interfacial fracture occurred. The stress–strain curves of the 3C- and 4H-SiC/Al interfaces in the C-top configuration exhibited ultimate tensile strengths of 6.603 GPa and 6.851 GPa, respectively, while their corresponding failure strains were 10% and 12%. These results indicate that the 4H-SiC/Al interface possesses superior overall mechanical properties. Analysis of the evolution of atomic relative positions and valence electron density with strain showed that fracture in all SiC/Al interfaces occurred within the Al matrix. Specifically, fracture in the 4H-SiC/Al interface occurred between the first and second Al atomic layers (Al1–Al2), in contrast to the 3C-SiC/Al interfaces, which fractured between the second and third Al layers (Al2–Al3). This distinction reflects the influence of different interfacial configurations on the bonding strength between aluminum atomic layers. The interfaces considered in this study are ideal, without defects or impurities. In contrast, real composite interfaces contain such features, which influence their mechanical behavior.

In summary, through a systematic evaluation of the atomic structure, tensile deformation behavior, and failure mechanisms of the 3C- and 4H-SiC/Al interfaces, this study provides a theoretical foundation and structural design guidelines for enhancing the mechanical properties of aluminum matrix composites via interface engineering. A valuable future direction would be to include temperature effects through ab initio molecular dynamics for a more accurate modeling of service conditions.

## Figures and Tables

**Table 1:** Comparison of calculated lattice constants of Al and SiC with existing theoretical and experimental data.

	Methods	Lattice Parameters		
		a (Å)	b (Å)	c (Å)
Al	Present study (GGA-PBE)	4.045	4.045	4.045
	Exp [27]	4.050	4.050	4.050
	Calc [18] (GGA-PBE)	4.044	4.044	4.044
	Calc [28] (GGA-PW91)	4.050	4.050	4.050
3C-SiC	Present study (GGA-PBE)	4.379	4.379	4.379
	Exp [29]	4.369	4.369	4.369
	Calc [30] (GGA-PBE)	4.366	4.366	4.366
	Calc [31] (GGA-PW91)	4.348	4.348	4.348
4H-SiC	Present study (GGA-PBE)	3.095	3.095	10.121
	Exp [32]	3.079	3.079	10.254
	Calc [30] (GGA-PBE)	3.084	3.084	10.096
	Calc [33] (LDA-CAPZ)	3.089	3.089	10.113

**Table 2:** Relative change in interlayer spacing after relaxation of the 3C-SiC (111) surface: effects of termination and slab thickness.

**Termination**	**Change in Interlayer**	**Slab Thickness**									
		**5**		**7**		**9**		**11**		**13**	
		**Si-III**	**Si-I**	**Si-III**	**Si-I**	**Si-III**	**Si-I**	**Si-III**	**Si-I**	**Si-III**	**Si-I**
Si	Δ*d*_12_ (%)	2.01	−2.20	1.42	−20.7	0.950	−19.6	0.74	−19.6	0.58	−19.1
	Δ*d*_23_ (%)	−8.85	4.48	−4.43	2.85	−3.15	2.40	−1.70	2.27	−1.58	2.16
	Δ*d*_34_ (%)			1.74	−5.06	1.05	−3.80	0.74	−3.48	0.53	−3.16
	Δ*d*_45_ (%)					−2.69	1.16	−1.90	1.05	−1.74	0.95
	Δ*d*_56_ (%)							0.74	1.74	0.42	−1.74
	Δ*d*_67_ (%)									−1.42	0.53
Termination	Change in interlayer	Slab thickness									
		5		7		9		11		13	
		C-III	C-I	C-III	C-I	C-III	C-I	C-III	C-I	C-III	C-I
C	Δ*d*_12_ (%)	0.95	−49.7	1.27	−42.6	1.21	−42.9	1.16	−42.9	1.11	−42.9
	Δ*d*_23_ (%)	−13.1	11.3	−1.27	5.17	−0.16	5.29	0.16	5.60	0.16	5.64
	Δ*d*_34_ (%)			1.53	−7.74	0.31	−6.31	0.16	−6.16	0	−5.84
	Δ*d*_45_ (%)					−0.94	−0.89	0.31	0.90	0	1.00
	Δ*d*_56_ (%)							0.21	−0.31	0	−0.16
	Δ*d*_67_ (%)									0.63	0.21

**Table 3:** Relative change in interlayer spacing after relaxation of the 4H-SiC(0001) surface: effects of termination and slab thickness.

**Termination**	**Change in Interlayer**	**Slab Thickness**									
		**9**		**11**		**13**		**15**		**17**	
		**Si-III**	**Si-I**	**Si-III**	**Si-I**	**Si-III**	**Si-I**	**Si-III**	**Si-I**	**Si-III**	**Si-I**
Si	Δ*d*_12_ (%)	0.89	−9.0	0.31	−8.03	0.68	−7.06	0.16	−7.38	0.53	−7.22
	Δ*d*_23_ (%)	−2.53	2.36	−1.12	2.05	−1.58	2.05	−0.64	1.89	−1.11	1.89
	Δ*d*_34_ (%)	0.73	−3.32	0.53	−2.37	0.42	−2.37	0.37	−2.06	0.37	−2.06
	Δ*d*_45_ (%)	−2.40	1.00	−1.42	0.84	−1.28	0.74	−0.95	0.63	−0.64	0.58
	Δ*d*_56_ (%)			0.37	−3.01	0.263	−1.28	0.21	−0.96	0.21	−0.80
	Δ*d*_67_ (%)					−1.27	0.31	−0.80	0.21	−0.79	0.21
	Δ*d*_78_ (%)							0.21	−0.79	0.26	−0.79
Termination	Change in interlayer	Slab thickness									
		9		11		13		15		17	
		C-III	C-I	C-III	C-I	C-III	C-I	C-III	C-I	C-III	C-I
C	Δ*d*_12_ (%)	1.16	−38.3	0.79	−44.9	1.11	−45.1	0.68	−45.1	1.05	−44.9
	Δ*d*_23_ (%)	0.96	5.51	0.31	5.67	1.44	5.67	−0.16	5.61	1.28	5.72
	Δ*d*_34_ (%)	0.52	−11.2	0.26	−9.63	0.16	−9.47	0.05	−9.63	0.05	−9.63
	Δ*d*_45_ (%)	−1.10	1.16	0.80	1.21	0.47	1.16	0.80	1.10	0.47	1.26
	Δ*d*_56_ (%)			0.37	−0.16	−42.6	−0.47	0.05	0.96	−0.05	−0.31
	Δ*d*_67_ (%)					0.48	0.26	0.79	0.05	0.48	0.31
	Δ*d*_78_ (%)							0.05	−0.79	0	0.64

**Table 4:** Calculated surface energies for various Al crystal planes.

Al_surf_	Al(001)	Al(110)	Al(111)
*E*_surf_ (J/m^2^)	0.954	1.035	0.825
Calc [36]	0.903	1.044	0.839
Calc [20]	1.024	1.073	0.936

**Table 5:** Calculated parameters (*W*_sep_ and *d*_0_) for 3C-SiC(111)/Al(111) interfaces.

Termination	Unrelaxed		Relaxed	
	*d*_0_ (Å)	*W*_sep_ (J/m^2^)	*d*_0_ (Å)	*W*_sep_ (J/m^2^)
C-top-SiC(111)/Al(111)	2.000	3.427	1.984	3.640
C-center-SiC(111)/Al(111)	2.000	2.540	1.983	2.711
C-hollow-SiC(111)/Al(111)	2.000	2.424	1.659	2.536
Si-top-SiC(111)/Al(111)	2.750	2.439	2.539	2.513
Si-center-SiC(111)/Al(111)	2.500	2.569	2.261	2.584
Si-hollow-SiC(111)/Al(111)	2.500	2.480	2.308	2.537

**Table 6:** Calculated parameters (*W*_sep_ and *d*_0_) for 4H-SiC(0001)/Al(111) interfaces.

Interface	Unrelaxed		Relaxed	
	*d*_0_ (Å)	*W*_sep_ (J/m^2^)	*d*_0_ (Å)	*W*_sep_ (J/m^2^)
C-top-SiC(111)/Al(111)	2.000	3.855	1.988	3.971
C-center-SiC(111)/Al(111)	2.000	3.347	1.985	3.581
C-hollow-SiC(111)/Al(111)	1.875	3.561	1.713	3.751
Si-top-SiC(111)/Al(111)	2.500	3.221	2.533	3.422
Si-center-SiC(111)/Al(111)	2.500	3.042	2.294	3.103
Si-hollow-SiC(111)/Al(111)	2.500	3.162	2.288	3.275

**Table 7:** Mulliken population analysis of atoms for the C- and Si-top terminations of the 3C-SiC(111)/Al(111) interface.

Type	Atoms	s	p	d	Total	Charge	Populations
C-top	Al	1.01	1.70	0.00	2.72	+0.28	0.25
	C	1.45	3.80	0.00	5.25	−1.25	
Si-top	Al	1.16	1.89	0.00	3.05	−0.05	0.33
	Si	1.08	1.99	0.00	3.07	+0.93	

**Table 8:** Mulliken population analysis of atoms for the C- and Si-top terminations of the 4H-SiC(0001)/Al(111) interface.

Type	Atoms	s	p	d	Total	Charge	Populations
C-top	Al	1.02	1.70	0.00	2.72	+0.28	0.26
	C	1.45	3.80	0.00	5.25	−1.25	
Si-top	Al	1.15	1.89	0.00	3.05	−0.05	0.35
	Si	1.09	1.98	0.00	3.07	+0.93	

**Table 9:** Comparison of work of separation, ultimate tensile strength, and ultimate tensile strain for the 3C- and 4H-SiC/Al interfaces.

Interface	Methods	Work of Separation (J/m^2^)	Ultimate Tensile Strength (GPa)	Ultimate Tensile Strain (%)
3C-SiC(111)/Al(111)	Present study (GGA-PBE)	3.640 (C-top)	6.603 (C-top)	10 (C-top)
4H-SiC(0001)/Al(111)	Present study (GGA-PBE)	3.971 (C-top)	6.851 (C-top)	12 (C-top)
3C-SiC(111)/Al(111)	Calc [19] (GGA-PBE)	3.576 (C-top)	6.33 (C-top)	10 (C-top)
4H-SiC(0001)/Al(111)	Calc [18] (GGA-PBE)	3.44 (C-top)	-	-
6H-SiC(0001)/Al(111)	Calc [40] (GGA-PBE)	5.09 (C-top)	3.90 (C-top)	10 (C-top)
6H-SiC(0001)/Al(111)	Calc [20] (GGA-PBE)	2.689 (C-top)	5.60 (C-top)	9 (C-top)
6H-SiC(0001)/Al(111)	Calc [41] (GGA-PBE)	-	4.27 (C-top)	11 (C-top)

**Table 10:** Mechanical properties of SiC/Al composites.

Materials	Yielding Strength (MPa)	Ultimate Tensile Strength (MPa)
9vol%SiCp/2009Al [42]	346.3 ± 3.0	≈510
17vol%SiCp/2009Al [43]	141.1	214.4
25vol%SiCp/2009Al [42]	395.8 ± 3.2	≈550

## Data Availability

The original contributions presented in this study are included in the article. Further inquiries can be directed to the corresponding authors.
